# Systematic analysis of lysine malonylation in *Streptococcus mutans*

**DOI:** 10.3389/fcimb.2022.1078572

**Published:** 2022-11-28

**Authors:** Zhengyi Li, Qinrui Wu, Yixin Zhang, Xuedong Zhou, Xian Peng

**Affiliations:** ^1^ State Key Laboratory of Oral Diseases, National Clinical Research Center for Oral Diseases, West China Hospital of Stomatology, Sichuan University, Chengdu, China; ^2^ Department of Cariology and Endodontics, West China Hospital of Stomatology, Sichuan University, Chengdu, China

**Keywords:** malonylation, post-translational modification, biofilm, *Streptococcus mutans*, bacteria, proteomics

## Abstract

Protein lysine malonylation (Kmal) is a novel post-translational modification (PTM) that regulates various biological pathways such as energy metabolism and translation. Malonylation in prokaryotes, however, is still poorly understood. In this study, we performed a global Kmal analysis of the cariogenic organism *Streptococcus mutans* by combining antibody-based affinity enrichment and high-performance liquid chromatography-tandem mass spectrometry (HPLC-MS/MS) analysis. Altogether, 392 malonyllysine sites in 159 proteins were identified. Subsequent bioinformatic analysis revealed that Kmal occurs in proteins involved in various metabolic pathways including translation machinery, energy metabolism, RNA degradation, and biosynthesis of various secondary metabolites. Quantitative analysis demonstrated that Kmal substrates were globally altered in the biofilm growth state compared to the planktonic growth state. Furthermore, a comparative analysis of the lysine malonylome of our study with previously determined lysine acetylome in *S. mutans* revealed that a small proportion of Kmal sites overlapped with acetylated sites, whereby suggesting that these two acylations have distinct functional implications. These results expand our knowledge of Kmal in prokaryotes, providing a resource for researching metabolic regulation of bacterial virulence and physiological functions by PTM.

## Introduction

During the past decade, various PTMs have been detected and characterized in prokaryotes as there have been advancements in high-quality antibodies and high sensitivity mass spectrometry. However, their functional and structural determination are particularly challenging, as most PTMs occur in relatively low number of prokaryotic proteins compared to eukaryotic proteins, and most of modified proteins have low levels of sub-stoichiometric modification (Macek et al., 2019). Protein phosphorylation, acetylation, succinylation, glycosylation, lipidation and pupylation are common PTM types in bacteria. These PTMs play vital roles in various cellular processes, including protein synthesis, carbon and nitrogen metabolism, cell cycle, persistence, and virulence. There are various PTMs on lysine residues, such as acetylation, crotonylation, and 2-hydroxyisobutyrylation, suggesting complicated regulatory mechanisms of protein functions (Hirschey and Zhao, 2015; Huang et al., 2020). Among these modifications, acetylation and succinylation are the most ubiquitous PTMs in bacteria, with global proteomic studies have reported that the numbers of acetylated and succinylated proteins typically surpass the numbers of proteins modified by other PTMs (Macek et al., 2019). However, the functional roles of these PTMs and the enzymes responsible for their attachment (writer) and removal (eraser) remain to be extensively studied.Lysine malonylation is an evolutionarily conserved PTM from bacteria to mammals and involves malonyl-coenzyme (CoA) as a cofactor. Since the malonyl group has an acidic carboxylic group under physiological pH, malonylated lysine is negatively charged, which might impact on protein function and enzymatic activities (Peng et al., 2011). Like other short-chain acyl-CoAs, malonyl-CoA can be synthesized from its corresponding short-chain acyl salt, malonate, catalyzed by malonyl-CoA synthetase (Witkowski et al., 2011). In addition, malonyl-CoA can be produced during the carboxylation of acetyl-CoA by acetyl-CoA carboxylase (ACC), the carboxylation of acetyl-CoA by propionyl-CoA carboxylase, and the β-oxidation of old-chain-length dicarboxylic acids (Peng et al., 2011). Most studies on Kmal have been performed in mammalian systems and identified thousands of malonylated sites, revealing its role in the progress of diseases like type 2 diabetes, schizophrenia, and cardiac hypertrophy (Du et al., 2015; Smith et al., 2022; Wu et al., 2022). There has been increasing interest in dissecting the regulatory roles of Kmal in several bacterial species, such as *Escherichia coli* (Qian et al., 2016), *Bacillus amyloliquefaciens* (Fan et al., 2017), *Mycobacterium tuberculosis* (Bi et al., 2022), and *Staphylococcus aureus* (Shi et al., 2021), which demonstrates that Kmal exists in diverse prokaryotic organisms and participates in the regulation of various physiological processes. Even so, whether Kmal exists and affects protein functions in streptococci remains unknown.

*S. mutans* is considered to be the most prevalent and cariogenic species in active carious lesions of humans, residing primarily in dental plaque, which is a biofilm that forms on the tooth surfaces. During the past decades, studies of *S. mutans* have focused on revealing the molecular mechanisms underlying the robust biofilm formation on tooth surfaces, the metabolism of a wide variety of carbohydrates obtained from the host diet, and the adaption of numerous environmental challenges (Lemos et al., 2019). Several studies have been performed to reveal the roles of PTMs in biofilm and cariogenic virulence. Wang et al. found that the phosphorylation of the response regulator of the two-component system VicRK could inhibit the expression of the glucosyltransferases GtfB and GtfC, thereby reducing the synthesis of extracellular polysaccharides (EPS), the major component of cariogenic biofilm (Wang et al., 2021). Acetylome study on the *S. mutans* depicted that acetylated substrates were globally altered in the biofilm state compared to the planktonic state, and the acetylated GtfB and C showed decreased activities (Lei et al., 2021; Ma et al., 2021). Our previous study revealed that the S-glutathionylation of a thioredoxin-like protein is important for interspecies competition and cariogenicity of *S. mutans* (Li et al., 2020). These results suggest that various PTM types exist in *S. mutans* and participate in diverse physiological processes. The genome of *S. mutans* UA159 encodes an acetyl coenzyme A carboxylase (ACC), which could synthesize malonyl-CoA through the carboxylation of acetyl-CoA (Ajdić et al., 2002), implying the existence of malonylation in this bacterium.

Therefore, this study aimed to confirm the existence of Kmal in *S. mutans* and identify the malonylated sites globally. The present findings provide a systematic view of the functional roles of Kmal in various metabolic pathways of *S. mutans*.

## Materials and methods

### Bacterial strain and growth conditions

*Streptococcus mutans* serotype c (strain ATCC 700610/UA159) was obtained from the Oral Microbiome Bank of China (Peng et al., 2020), and grown at 37 °C under an aerobic (5% CO2, 95% air) condition in brain heart infusion (BHI) broth (Difco, Sparks, MD, USA). For biofilm formation, the bacteria in the mid-exponential phase were inoculated into fresh BHI supplemented with 1% sucrose with a 1:100 dilution.

### Protein extraction

After 24h growing in the media with or without sucrose, the cells were collected by centrifugation and washed twice with PBS. Cell pellets were placed in ground liquid nitrogen to break the cell wall. Four times the volume of lysis buffer (8M urea, 1% proteinase inhibitor cocktail, 3 μM trichostatin A, 50 mM nicotinamide) was added to resuspend them. The samples were then sonicated and centrifuged (12000 × g at 4 °C for 10 min) for removing cellular debris. The supernatants were transferred to a new centrifuge tube, and the protein concentration was determined a with bicinchoninia acid (BCA) kit (Beyotime Biotechnology, JiangSu, China) according to the manufacturer’s instructions.

### Western blotting

Extracted proteins were standardized to the same concentration and boiled in SDS loading buffer for 5 min. Samples were then subjected to 11% SDS-PAGE and transferred to a nitrocellulose (NC) membrane. The membrane was blocked for 1 h in TBST buffer (25mM Tris-HCl, pH8.0, 150 mM NaCl, 0.1% Tween-20) supplemented with 5% defatted milk powder with further incubation overnight at 4°C with the pan anti-Kmal monoclonal antibody (cat. #PTM-902, PTM Bio Inc., Hangzhou) (1:1000, in TBST buffer with 5% defatted milk powder). After three consecutive washes with TBST buffer, the membrane was incubated with goat anti-mouse IgG antibody horseradish peroxidase conjugate (1:5000 in TBST buffer; Thermo Fisher Scientific, Waltham, MA, USA). After six times washing, an ECL substrate kit (Millipore) was used for protein visualization.

### Trypsin digestion

The same amount of protein from each sample was separated for digestion. One volume of precooled acetone was initially added to the samples. Four times the volume of acetone was then added after mixing thoroughly with a vortex and allowing them to precipitate at -20°C for 2 h. Then, centrifugation was performed for 5 min, and the supernatants were discarded. The precipitation was washed twice with precooled acetone. Next, 200 mM tetraethylammonium bromide (TEAB) was added to dry precipitation and sonicate for dispersion. Trypsin was added at a 1:50 (trypsin: protein) mass ratio and digested overnight at 37°C. The peptides were then reduced with 5mM dithiothreitol for 30 min at 56°C and alkylated with 11 mM iodoacetamide in darkness for 15min at room temperature.

### Enrichment of malonyated peptides

Tryptic peptides were dissolved in an immunoprecipitating (IP) buffer (100 mM NaCl, 1 mM EDTA, 50 mM Tris-HCl, 0.5% NP-40, pH 8.0) and incubated with pre-washed pan anti-Kmal antibody resins (cat. #PTM-904, PTM Bio Inc., Hangzhou) at 4°C, overnight, with gentle shaking in the dark. The resins were washed four times with an IP buffer and twice with deionized water. The bound peptides were eluted from the resins three times with 0.1% trifluoroacetic acid. Finally, the enriched peptides were desalted using C18 ZipTips (Millipore) and dried by vacuum.

### Peptides analysis by high-performance liquid chromatography-tandem mass spectrometry

The peptides were dissolved in solvent A (0.1% formic acid and 2% acetonitrile in water) and a NanoElute UPLC system (ThermoFisher Scientific) was used for separation. The gradient comprised 6%–22% solvent B (0.1% formic acid in 100% acetonitrile) in 0–40 min, 22%–30% in 40–52 min, 30%–80% in 52–56 min, climbing to 80% in 56–60 min, all at a constant flow rate of 450 nL/min. The eluted peptides were subjected to a capillary nanospray ionization (NSI) source followed by tandem mass spectrometry (MS/MS) in timesTOF Pro (Bruker Daltonics). The electrospray voltage applied was 1.65 kV. Precursors and fragments were analyzed using the TOF detector, with a MS/MS scan range of 100 to 1700 m/z. The timsTOF Pro tool was operated in parallel accumulation serial fragmentation (PASEF) mode. Precursors with charge states of 0 to 5 were selected for fragmentation, and 10 times of PASEF-MS/MS scans were acquired per cycle. The dynamic exclusion was set to 24 s.

### Database search

Fragmentation data were then processed using the Maxquant search engine (v.1.6.15.0) against the Blast *Streptococcus mutans* serotype C (UA159/ATCC700610) containing 1,953 sequences and concatenated with the reverse decoy database. Trypsin/P was specified as a cleavage enzyme, allowing up to four missing cleavages and 5 modifications per peptide. The mass error for precursor ions was set to 20 ppm for the first search and 20 ppm for the main search. The mass error for fragment ions was set to 20ppm. Peptides with a length of at least seven amino acid residues were used for further analysis. Carbamidomethyl Cys was specified as fixed modification, while acetylation of protein N-terminal, lysine malnoylation and methionine oxidation were specified as variable modifications. The maximum false discovery rate (FDR) threshold for proteins, peptides, and the spectrum was adjusted to < 1%.

### Motif analysis

All identified Kmal substrates in *S. mutans* were used to analyze the flanking sequences at sites of Kmal with Motif-X algorithms-based MoMo software. Ten neighboring amino acid residues on each side of the modification site were selected as the positive set. All genes of *Streptococcus mutans* serotype C (UA159/ATCC700610) amounted to the negative set.

### Protein annotation and functional enrichment

Protein annotation and functional enrichment were performed as previously described (Li et al., 2020). The Gene Ontology (GO) annotation proteome was derived from the UniProt-GOA database (http://www.ebi.ac.uk/GOA/). The domain functional descriptions were annotated by Pfamscan based on the protein sequence alignment method and the Pfam domain database (https://pfam.xfam.org/). The Kyoto Encyclopedia of Genes and Genomes (KEGG) database (https://www.kegg.jp/kegg/) was used to annotate the protein pathways. The prokaryotic organism subcellular localization prediction software CELLO was used to predict subcellular localization. The clusters of Orthologous Groups (COG) database (http://www.ncbi.nlm.nih.gov/COG) were used to align and classify the orthologs of proteins.

For each GO category, a two-tailed Fisher’s exact test was employed to test the enrichment of differentially expressed proteins against all the identified proteins. The GO terms with a corrected *P* < 0.05 were considered significant. The KEGG database was used to identify the enriched pathways by a two-tailed Fisher’s exact test and assess the enrichment of differentially expressed proteins against all the identified proteins. The pathways with a corrected *P* < 0.05 were considered significant. These pathways were classified into hierarchical categories according to the KEGG website. For each category of proteins, the Pfam database was researched, and a two-tailed Fisher’s exact test was employed to test the enrichment of differentially modified proteins against all the identified proteins. Protein domains with a corrected *P* < 0.05 were considered significant.

### Functional enrichment-based clustering

After functional enrichment, we collated all the categories and their *P* values. This was followed by filtering for categories that were at least significantly enriched (*P* < 0.05) in at least one of the clusters and transforming these P-value matrixes by the function X = –log10 (*P*-value). These X values were then Z-transformed for each functional category. These Z scores were finally clustered by one-way hierarchical clustering (Euclidean distance, average linkage clustering) in Genesis. The heatmap.2 function from the gplots R-package was applied to visualize the cluster membership.

### Protein-protein interaction network analysis

The STRING database (version 10.5) was used for the enrichment analysis of *S. mutans* Kla protein-protein interaction networks. Only high-confidence interactions (with a score > 0.7) in the STRING database were fetched for the analysis. The MCODE plug-in toolkit was used to identify highly connected clusters, and the interaction network was visualized by Cytoscape software version 3.7.2.

### Statistical analysis

For proteomic studies, the relative quantitative values of every site/protein in each group were performed with a t-test to assess for a significant difference. For all above enrichment methods, a Fisher’s exact test was employed to assess the enrichment of differentially expressed proteins against all the identified proteins. A difference was considered significant if *P* < 0.05.

## Results

### Lysine malonylome profiling in *Streptococcus mutans*

**Figure 1:**
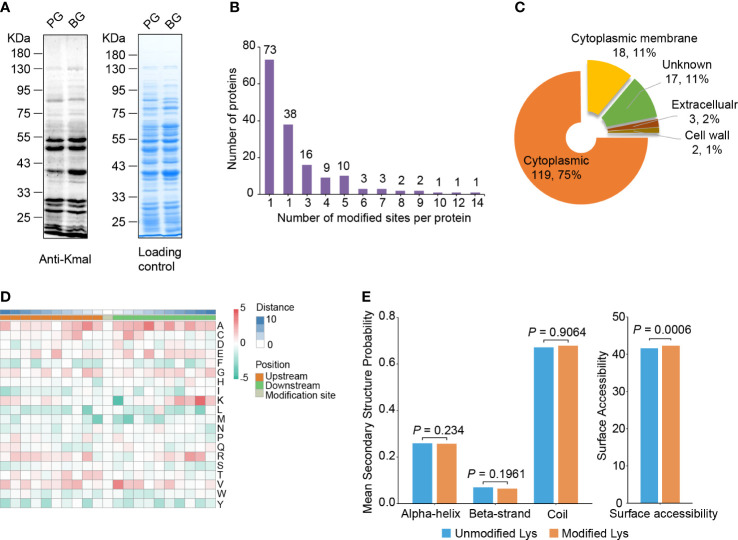
Lysine malonylome profiling in *S. mutans.* **(A)** Immunoblot analysis of malonylated proteins with pan anti-Kmal antibody in *S. mutans* from planktonic growth (PG) and biofilm growth (BG). Coomassie brilliant blue R-250 staining of SDS-PAGE gel as the loading control. **(B)** The number of malonylated sites identified per protein in *S. mutans*. **(C)** Subcellular localization prediction of modified proteins. **(D)** Motif analysis of all identified Kmal proteins. Red indicates high frequency and green means low frequency. **(E)** Distribution of Kmal sites in different secondary structures (left panel) and their surface accessibility (right panel). The *P* values were calculated with the Wilcoxon test.

To demonstrate the existence of Kmal in *S. mutans* and evaluate the alterations of Kmal in different growth states, we first compared the Kmal levels of *S. mutans* cells in biofilm growth (BG) with those in planktonic growth (PG) using a pan anti-Kmal antibody (**Figure 1A**). Multiple protein bands spanning a wide mass range were detected, and the Kmal levels of some proteins were significantly changed between these growth conditions. To elucidate Kmal in *S. mutans* systematically, we performed a quantitative proteomic analysis of Kmal substrates using by combining antibody-based affinity enrichment and high-resolution LC-MS/MS analysis. A total of 392 malonylated residues of 392 peptides were identified in 159 proteins using a highly conservative threshold (FDR < 1%) (**Supplementary Table 1**, **Supplementary Figure 1**). Among these modified proteins, 45.9% were modified in only one site, while only 8.2% were modified in six or more sites (**Figure 1B**). The most heavily malonylated proteins included putative bacitracin synthetase 1 (14 sites), pyruvate kinase (12 sites), and chaperone protein DnaK (10 sites). Moreover, the proportion of malonylated proteins among the total proteins of *S. mutans* UA159 was about 8.1% (159/1953); these proportions for in *E. coli* (Qian et al., 2016) was 13.8% (594/4306), 10.2% (382/3728) in *B. amyloliquefaciens* (Fan et al., 2017), 25.7% (1026/3993) in *M. tuberculosis* (Bi et al., 2022), and 9.7% (281/2889) in *S. aureus* (Shi et al., 2021).

PSORTb software was used to predict and annotate the subcellular localization of the manoylated proteins (Yu et al., 2010). The results showed that most of the modified proteins (119) were cytoplasmic, accounting for 74.8% of the total modified proteins (**Figure 1C**). Eighteen malonylated proteins were predicted to reside in the cytoplasmic membrane, these proteins might be related to transmembrane transportation, bacterial adhesion, and resistance to fluctuating environments, for example, the PTS system sucrose-specific EIIBCA component, which is responsible for the internalization of sucrose. In addition, several proteins were located outside of the cell or on the cell wall, involving the glucosyltransferase GtfC and the levansucrase Ftf, suggesting that Kmal is related to EPSs synthesis and biofilm formation (**Table 1**).

**Table 1:** The malonylated proteins that are located in the cytoplasmic membrane, cell wall or extracellular.

Subcellular localization	Proteinaccession	Gene name	Protein description	Modified sites
**Cytoplasmic membrane**	I6L922	*SMU_765*	NADH oxidase/alkyl hydroperoxidase reductase peroxide-forming	109
	P12655	*scrA*	PTS system sucrose-specific EIIBCA component	46, 601
	Q8CVC6	*prsA*	Foldase protein PrsA	253
	Q8DRU8	*opuCa*	Putative osmoprotectant amino acid ABC transporter, ATP-binding protein	23
	Q8DRV8	*SMU_2104*	APC family permease(predicted)	472
	Q8DS76	*SMU_1957*	Putative PTS system, mannose-specific IID component	162, 211
	Q8DSQ4	*SMU_1719c*	UPF0154 protein	59
	Q8DSX5	*pfs*	5’-methylthioadenosine/S-adenosylhomocysteine nucleosidase	37
	Q8DSZ9	*SMU_1602*	Putative NAD(P)H-flavin oxidoreductase	109
	Q8DT86	*SMU_1479*	DUF3042 family protein(predicted)	31, 40
	Q8DTJ2	*SMU_1345c*	Putative peptide synthetase	28, 123, 121, 355
	Q8DTJ4	*SMU_1343c*	Putative polyketide synthase	321
	Q8DTL0	*ftsE*	Cell division ATP-binding protein FtsE	130
	Q8DV71	*SMU_635*	VIT family protein(predicted)	105, 96
	Q8DVD4	*divIVA*	Putative cell division protein DivIVA	108, 110, 133, 221
	Q8DVD9	*ftsZ*	Cell division protein FtsZ	65, 320
	Q8DVE5	*bipA*	50S ribosomal subunit assembly factor BipA	544
	Q8DW45	*ilvB*	Acetolactate synthase	169
**Cell wall**	P11000	*wapA*	Wall-associated protein	184
	P23504	*spaP*	Cell surface antigen I/II	102, 184, 1002, 1157
**Extracellular**	P13470	*gtfC*	Glucosyltransferase-SI	156, 870, 928
	P11701	*ftf*	Levansucrase	105
	Q8DWM3	*gbpB*	Putative secreted antigen GbpB/SagA putative peptidoglycan hydrolase	105, 221

To assess the conserved substrate motifs of malonylated residues, we analyzed the adjacent amino acids of the malonylated lysine from -10 to +10 using Motif-X algorithms (**Figure 1D**) (Schwartz and Gygi, 2005). The results revealed that the aliphatic amino acids alanine and valine were overrepresented in the region flanking Kmal sites. Meanwhile, the sulfur-containing amino acid methionine and the aromatic phenylalanine, tryptophan and tyrosine were underrepresented. These results differed from the study on Kac in *S. mutans* (Lei et al., 2021), indicating that these two lysine modifications have distinct sequence alignments.

Furthermore, secondary structure distributions and surface accessibility of malonylated residues were analyzed using the NetSurP algorithm (Høie et al., 2022). The distribution pattern of Kmal exhibited no significant difference from that of non-modified lysine residues, which suggests that there was no structural preference for Kmal in *S. mutans* (**Figure 1E**). The average surface accessibility of malonylated lysine residues was significantly higher (*P* = 0.0006) than that of unmodified lysine, indicating that Kmal is preferably located on the surface of the protein structure. These results were similar to those of a previous study on Kmal in *Staphylococcus aureus* (Shi et al., 2021).

### Functional analysis of the malonylome in *Streptococcus mutans*

**Figure 2:**
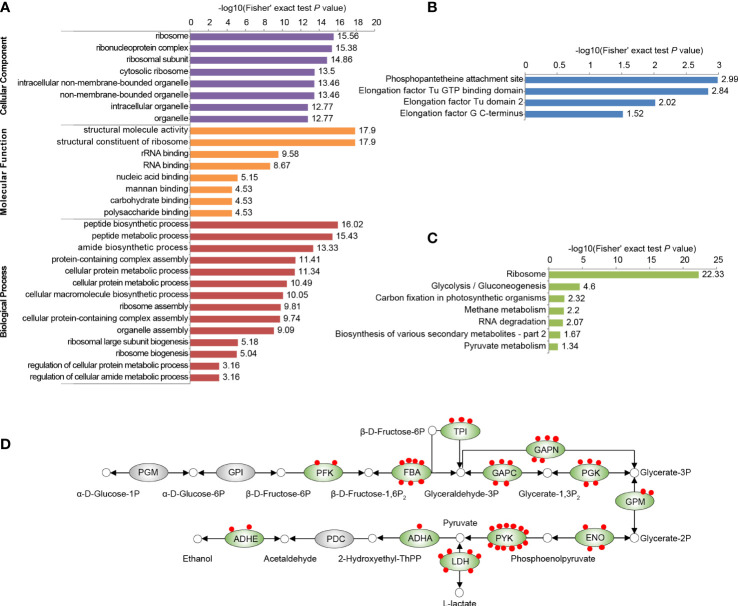
Functional enrichment analysis of all identified Kmal substrates in our proteomic study. **(A)** GO-based enrichment analysis of malonylated proteins. **(B)** Pfam-based enrichment analysis of domains related to malonylated proteins. **(C)** KEGG pathway-based enrichment analysis of malonylated proteins. For each category, a two-tailed Fisher’s exact test was employed to test the enrichment of the identified modified proteins against all proteins in the species database. Fold enrichment > 1.5 and adjusted *P* values < 0.05 were considered significant. **(D)** Malonylated enzymes involved in glycolysis in *S. mutans*. Green ovals indicate proteins subjected to malonylation, red dots represent Kmal sites. PGM, Phosphoglycerate mutase; GPI, glucose-6-phosphate isomerase; PFK, ATP-dependent 6-phosphofructokinase; FBA, fructose-1,6-biphosphate aldolase; TPI, triosephosphate isomerase; GAPC, glyceraldehyde-3-phosphate dehydrogenase; GAPN, NADP-dependent glyceraldehyde-3-phosphate dehydrogenase; PGK, phosphoglycerate kinase; GPM, glucosephosphate-mutase; ENO, enolase; PYK, pyruvate kinase; LDH, L-lactate dehydrogenase; ADHA, putative acetoin dehydrogenase (TPP-dependent), E1 component alpha subunit; PDC, pyruvate decarboxylase; ADHE, Aldehyde-alcohol dehydrogenase.

To investigate the function of Kmal in regulating the cellular physiological processes of *S. mutans*, we conducted functional enrichment analyses of the Kmal substrates that we identified. GO-based enrichment (**Figure 2A**) showed that malonylated proteins were mostly enriched in protein expression-related processes, with specific enrichment in the peptide biosynthetic process (*P* = 9.6 × 10^-17^), the peptide metabolic process (*P* = 3.7 × 10^-16^), and the amide biosynthetic process (*P* = 4.7 × 10^-14^). The molecular functions of Kmal substrates were mainly enriched in the structural molecule activity (*P* = 1.3 × 10^-18^) and the structural constituent of ribosome (*P* = 1.3 × 10^-18^). Correspondingly, many modified proteins were primarily located in the ribosome (*P* = 2.8 × 10^-16^). Moreover, domain analysis based on the Pfam database (**Figure 2B**) revealed that Kmal substrates were significantly enriched in the functional domains of elongation factors, which are vital for the elongation of peptides during the translation process, suggesting a role of Kmal in protein synthesis in *S. mutans*. Interestingly, the phosphopantetheine attachment site was the most significantly enriched term in this analysis (*P* = 1.0 × 10^-3^), and two acyl carrier proteins (Q8DSN3, Q8DWL8) were involved, indicating that Kmal might play a functional role in regulating fatty acid or polyketide biosynthesis.

According to KEGG-based metabolic pathway enrichment (**Figure 2C**), the top enriched pathways were ribosome (*P* = 4.6 × 10^-23^) and glycolysis/gluconeogenesis (*P* = 2.5 × 10^-5^). Glycolysis/gluconeogenesis is the key energy metabolic pathway of the facultative aerobic *S. mutans*. A total of 55 Kmal sites were identified on 12 glycolytic enzyme (**Figure 2D**), among which pyruvate kinase (PYK) harbored 12 Kmal sites, while fructose-1,6-biphosphate aldolase (FBA) and L-lactate dehydrogenase (LDH) harbored seven and six Kmal sites, respectively. These functional enrichment results suggested that Kmal has profound effects on various vital biological pathways by regulating the functions of many enzymes.

**Figure 3:**
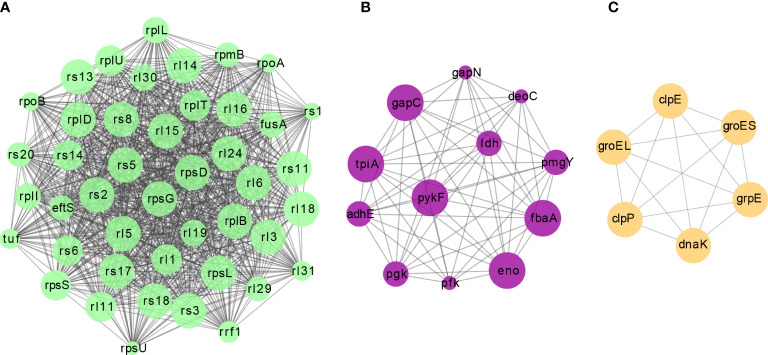
The top three clusters of highly interconnected malonylated PPI networks. Interaction network of lysine-lactylated proteins (listed in gene names) were analyzed using the MCODE plug-in toolkit in the Cytoscape software (version 3.7.2). **(A)** Cluster 1: MCODE score = 42.186, nodes = 44, edges = 907. **(B)** Cluster 2: MCODE score = 11.091, nodes = 12, edges = 61. **(C)** Cluster 3: MCODE score = 6, nodes = 6, edges = 15.

Protein-protein interactions (PPI) are vital for biochemical reactions and vulnerable to PTMs (Yao et al., 2019). The interactions between all identified malonylated proteins in this study were mapped using the STRING database (Szklarczyk et al., 2021). Combining cluster analysis with the MCODE module in Cytoscape software characterized nine highly interconnected networks (**Supplementary Table 2**). We visualized the top three enriched interaction clusters from the analysis (**Figure 3**). Interestingly, almost all proteins in cluster 1 are ribosome- associated proteins (**Figure 3A**), while most proteins in cluster 2 are glycolytic enzymes (**Figure 3B**). These results are aligned with the KEGG pathway enrichment conducted. Taken together, Kmal in *S. mutans* might potentially impact the PPIs, thus contributing to the regulation of metabolic pathways.

### Lysine malonylome in biofilm is significantly different from that of planktonic growth

**Figure 4:**
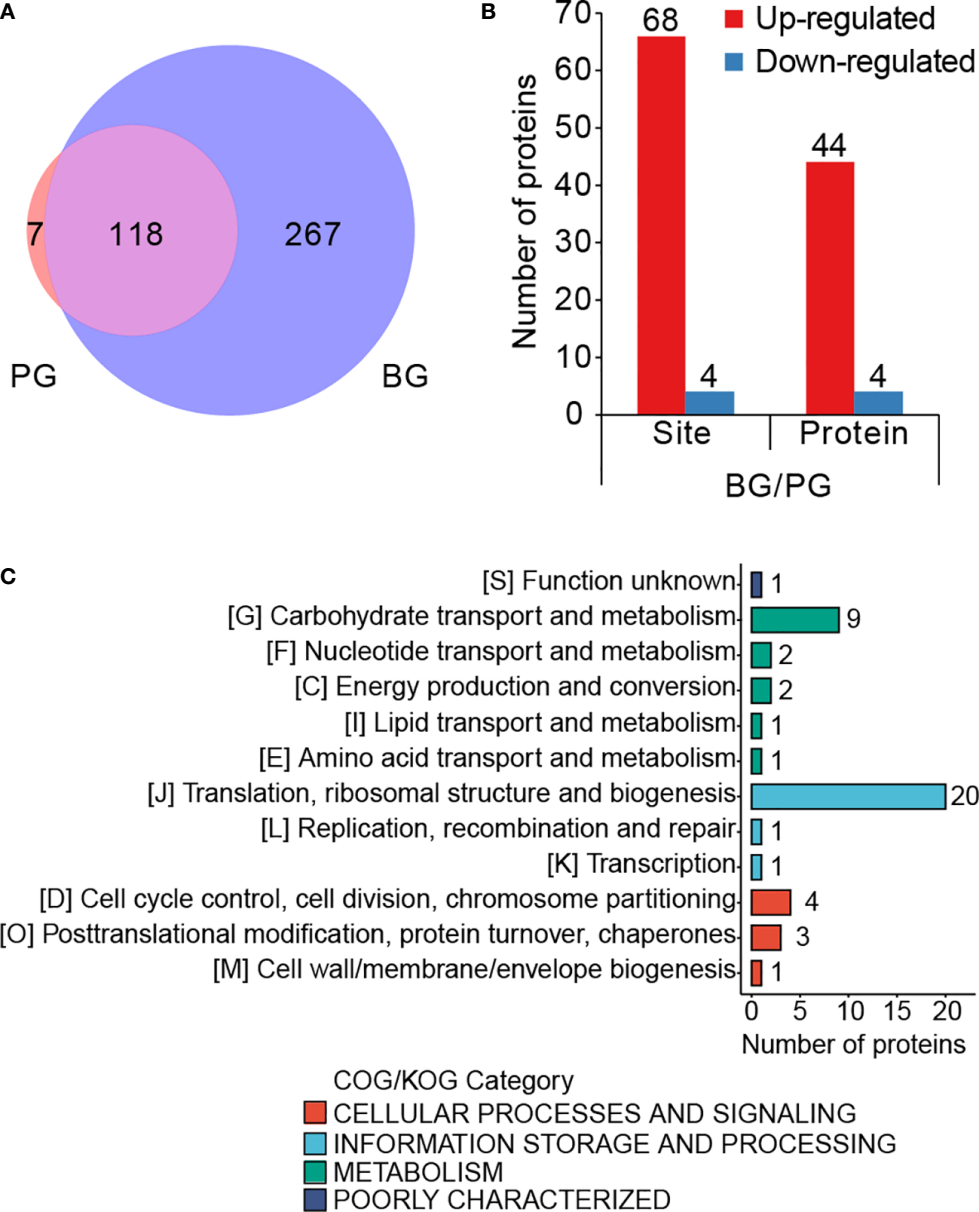
Lysine malonylome in biofilm is significantly different from that of planktonic growth. **(A)** Venn diagram showing the total number of PG-only (~1.79%), BG-only (~68.11%) and overlapping (~30.1%) modified sites (filtered with a threshold value of modification fold change >1.5 or <1.5). **(B)** Bar graph for the number of differentially modified sites and the corresponding proteins. **(C)** COG-based classification of the differentially modified proteins.

Cariogenic biofilms are vital for develop ing dental caries (Lemos et al., 2019). Thus, we investigated the role of Kmal in biofilm formation by comparing the lysine malonylome of *S. mutans* cells from biofilm (BG group) with that of planktonic cells (PG group). Of all the identified Kla sites in these two groups, 118 sites were present in cells from both groups, only seven sites were detected only in the planktonic cells, whereas 267 sites were present only in the cells from biofilm (**Figure 4A**). Subsequent quantitative analysis depicted that 117 residues from 66 proteins could be quantified in both groups using MaxQuant software (**Supplementary Figure 1**, **Supplementary Table 1**). We examined the change in these quantifiable sites in the BG group relative to the PG group. Finally, the modification levels of 66 lysine residues on 44 proteins were upregulated, but only four sites on four proteins were downregulated (**Figure 4B**) (filtered with a threshold value of modification fold change >1.5 or <1.5) (**Supplementary Table 3**). Additionally, since 68.1% of the malonylome (267 of 392 sites) is present in only the BG group, the increase in Kmal is more vigorous than the quantitative analysis.

To investigate the functional roles of these differentially modified proteins during biofilm formation, we performed functional analyses of these proteins corresponding to the differentially modified sites. Classification based on prokaryotic orthologous groups (COG) revealed that the differentially modified proteins broadly participated in translation, ribosomal structure and biogenesis, carbohydrate transportation, and metabolism (**Figure 4C**). After that, we performed a cluster analysis of these differentially modified sites. The differentially modified sites were divided into four quorums based on the fold change (FC) values, namely Q1, 0< FC <0.5; Q2, 0.5 < FC < 1/1.5; Q3, 1.5 < FC < 2; Q4, FC > 2 (**Supplementary Figure 2A**). Functional enrichment analysis was conducted for these quorums (**Supplementary Figure 2B**). GO-based cluster analysis showed that the proteins with upregulated sites were enriched on the cell surface. Meanwhile, the proteins in Q4 were enriched in the response to heat, the symbiotic process, and the interspecies interaction between organisms. Meanwhile, the KEGG-based analysis showed that the proteins with increased modified sites were enriched in purine metabolism. These results suggested that Kmal might be an important tool for regulating the biological processes in biofilm for *S. mutans.*

### Comparison of acetylome and malonylome in *Streptococcus mutans*

**Figure 5:**
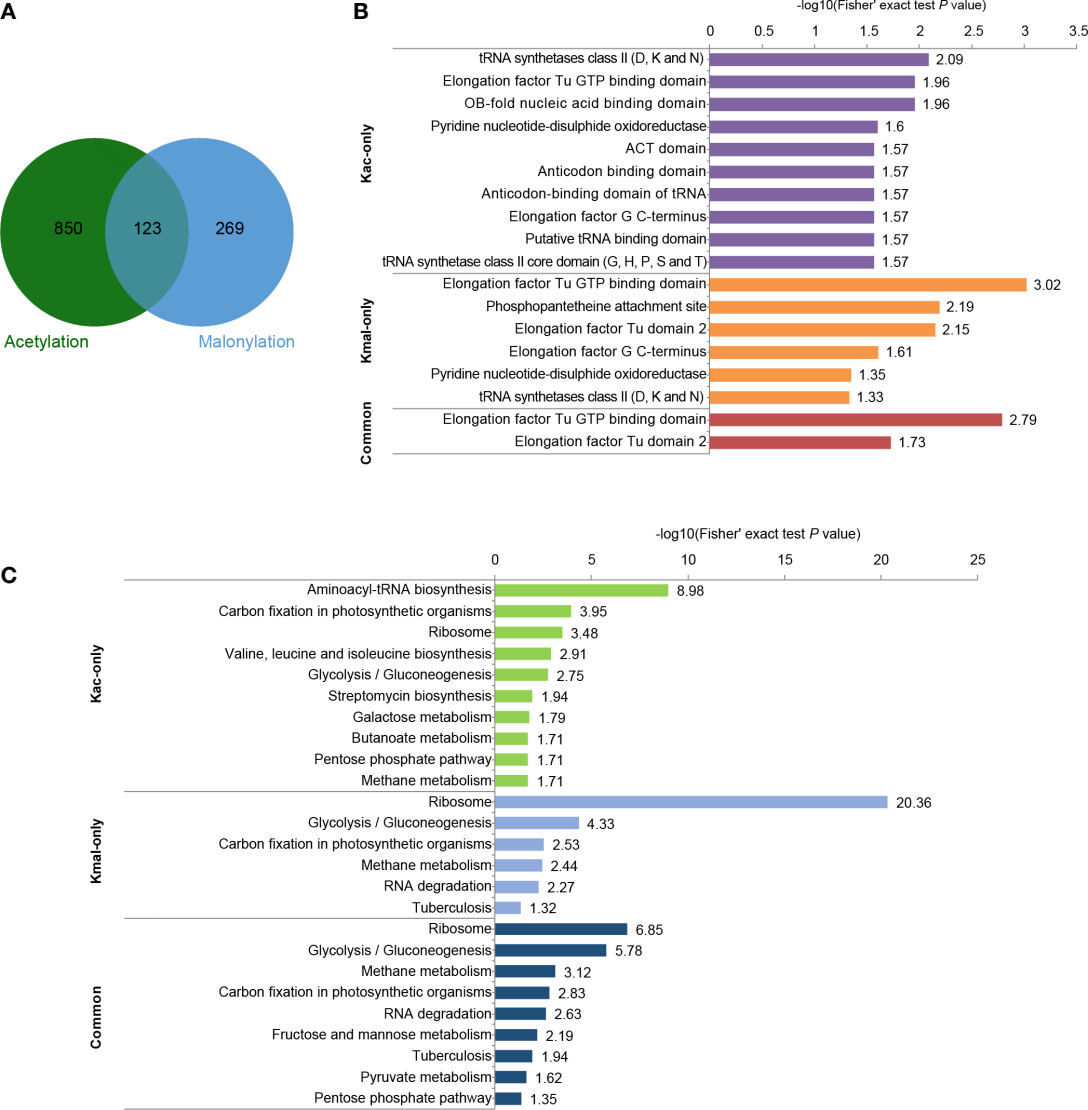
Comparison of acetylome and malonylome in *S. mutans*. **(A)** Venn diagram showing the total number of Kac-only, Kmal-only and overlapping sites. **(B)** Pfam-based domain enrichment for these common and unique sites of the two acylome datasets. **(C)** KEGG-based pathway enrichment for these common and unique sites of the two acylome datasets. For each category, a two-tailed Fisher’s exact test was employed to test the enrichment of the identified modified proteins against all proteins in the species database. Fold enrichment > 1.5 and adjusted *P* values < 0.05 were considered significant.

To investigate the similarities and differences among the various lysine acylation modifications, we further compared our malonylome dataset with a previously reported acetylome dataset in *S. mutans* UA159 (Lei et al., 2021). It was found that 31.4% of the malonylation sites could also be acetylated (**Figure 5A**, **Supplementary Table 4**). These two kinds of modifications are highly overlapping in several proteins, including pyruvate kinase (Q8DTX7, seven overlapping sites), chaperone protein DnaK (O06942, six overlapping sites), and L-lactate dehydrogenase (P26283, six overlapping sites). Moreover, 87.4% of Kac sites were identified in only the lysine malonylome dataset, and 68.6% of Kmal sites were identified in only the lysine acetylome dataset.

Afterward, performed functional analyses of these common and unique sites of the two acylome datasets. Pfam-based domain enrichment showed that the sites bearing either or both of the modifications broadly resided in the same domains, including the tRNA synthetases class II, the elongation factor Tu GTP-binding domain, and the elongation factor G C-terminus (**Figure 5B**). These domains are important for protein synthesis. In support of this observation, many proteins with either or both of these modifications were significantly enriched in ribosome based on the KEGG analysis (**Figure 5C**), whereby indicating that both of these modifications have functions in regulating protein expression in *S. mutans*. In addition, the glycolysis/gluconeogenesis pathway was also the common pathway enriched by both of these modifications. However, enrichment analysis also demonstrated that these two modifications were enriched in many different metabolic pathways. For example, the proteins with sites that were identified in only the Kac dataset depicted significant enrichment in pathways such as aminoacyl-tRNA biosynthesis, galactose metabolism and pentose phosphate, while the proteins bearing sites that were only modified by malonylation were enriched in methane metabolism and RNA degradation. These results suggested that Kmal could have distinct physiological roles from those of Kac.

For those sites identified in both Kmal and Kac datasets, their functional enrichment results showed significant enrichment in methane metabolism, RNA degradation, fructose and mannose metabolism and pyruvate metabolism, except for the pathways enriched by the unique sites of the two acylome datasets.

## Discussion

Our proteomic approach identified 392 lysine-malonylated sites on 159 proteins of *S. mutans*, generating the first malonylome dataset for streptococci. These malonylated proteins are distributed in various cellular components and participate in multiple crucial biological pathways. Compared with the bacteria growing in a planktonic state, the levels of malonylation in bacteria growing in a biofilm state have significantly improved, which suggests a crucial role of Kmal in regulating the physiological activities in cariogenic biofilm. Combining analysis with the acetylome dataset of *S. mutans*, we observed that a small proportion of acetylated sites could also be malonylated, suggesting that these two kinds of modification have connection or interaction in regulating the functions of protein. In conclusion, this study provides significant resources for further functional investigations into Kmal in bacteria.

Like other large-scale studies of malonylation in bacteria (Qian et al., 2016; Shi et al., 2021; Bi et al., 2022), our data revealed that many Kmal substrates were enriched in ribosomal and translation-associated proteins, but there is no report revealing the functional role of Kmal in translation. Few studies have investigated the impacts of PTMs on ribosomal proteins. In *E. coli*, Kac has been proven to promote the dissociation or inhibit the association of 30S and 50S ribosomal subunits, thus interfering with translation (Feid et al., 2022). Phosphoproteome profiling across ribosomal subcomplexes revealed that phosphorylation on the ribosomal protein RPL12/uL11 was strongly depleted in polysomes, with subsequent experiments demonstrating that this modification regulates the translation of mitosis-regulated mRNAs, thus contributing to the regulation of translation during mitosis (Imami et al., 2018). An investigation into UFMylation in human cells revealed that RPL26 UFMylation plays a direct role in cotranslational protein translocation into the endoplasmic reticulum (ER). The inhibition of RPL26 UFMylation leads to impaired ER protein homeostasis, suggesting that UFMylation of RPL26 has an impact on protein biogenesis in the early secretory pathway (Walczak et al., 2019). Moreover, the regulatory mechanisms of the modification status of ribosomal proteins have been dissected in several studies. For example, oligoglutamylation of the *E. coli* ribosomal protein S6 is carried out by the ATP-dependent glutamate ligase RimK (Kang et al., 1989; Kino et al., 2011; Zhao et al., 2013), which is also widespread and conserved in hundreds of prokaryotic and eukaryotic genomes (Little et al., 2016). In our quantitative analysis of Kmal in *S. mutans*, 20 of the 44 proteins with increased modified sites were involved in translational processes (**Figure 4C**), which implies that Kmal plays a vital role in regulating protein synthesis in cariogenic biofilm.

Reversible PTMs are tightly regulated through the addition and removal of chemical groups by regulatory enzymes. In mammalian cells, the lysine deacetylase SIRT5 was demonstrated to catalyze a lysine demalonylation reaction (Peng et al., 2011). Recently, the lysine acetyltransferase KAT2A showed lysine malonyltransferase activity in malonylation of the histone H2B_K5 (Zhang et al., 2022). However, no prokaryotic demalonylase or malonyltransferase has been reported as of yet. New reports on the characterization of lysine acetyltransferase (KAT) and deacetylase are appearing continuously, such as the KAT PatZ and the deacetylases CobB and YcgC from *E. coli* (Colak et al., 2013; Tu et al., 2015; Dash and Modak, 2021), the KAT Eis from *M. tuberculosis* (Ghosh et al., 2016), and the ActG from *S. mutans* (Ma et al., 2021). Interestingly, the deacetylase CobB also possesses a desuccinylase activity in *E. coli* (Colak et al., 2013), suggesting that the multifunctional roles of these identified acetyltransferase and deacetylase need to be investigated. Additionally, given that both acetylation and succinylation can also occur nonenzymatically (Wagner and Payne, 2013; Wolfe, 2016), efforts should be made to verify nonenzymatic malonylation in bacteria.

Kac neutralizes the positive charge on the side chain of a lysine residue, while Kmal adds a negative charge. Our comparative analysis showed that the same lysine residue could be either acetylated or malonylated. These two kinds of PTMs could have different effects on protein structure, PPIs, DNA–protein interactions, and enzymatic activities, thus contributing to bacterial rapid adaptation to environmental changes (Macek et al., 2019). As shown in **Figure 5**, these enrichment results of Kmal were to some extent similar to those of Kac. This similarity might be due to the common shared pathways among these two kinds of PTMs. It is also possibly cause by the intrinsic antibody-based enrichment approach bias toward the capture of modified peptides from abundant proteins. In addition, our enrichment analysis results were biased toward high abundant proteins as we did not take into consideration the information on the number of Kmal sites on each protein or Kmal peptide intensity. Therefore, these bioinformatics analysis results can only be used as a reference, and the detailed functional importance and mechanisms of Kmal in *S. mutans* needs to be further studied.

## Data availability statement

The MS proteomics data presented in the study are deposited in the PRIDE repository (https://www.ebi.ac.uk/pride), accession number PXD038045.

## Author contributions

XP, ZL and YZ conceived and designed the experiments. ZL, QW and YZ performed the experiments. ZL analyzed the data. YZ and XP further contributed reagents and materials tools. ZL wrote initial draft of the manuscript. All authors contributed to the article and approved the submitted version.

## Funding

This work is supported by grants (32070120, 81870754) from the National Natural Science Foundation of China.

## Acknowledgments

We thank the scientists from PTM BioLab (Hangzhou) for the assistance of omic analysis.

## Conflict of interest

The authors declare that the research was conducted in the absence of any commercial or financial relationships that could be construed as a potential conflict of interest.

## Publisher’s note

All claims expressed in this article are solely those of the authors and do not necessarily represent those of their affiliated organizations, or those of the publisher, the editors and the reviewers. Any product that may be evaluated in this article, or claim that may be made by its manufacturer, is not guaranteed or endorsed by the publisher.
