# Supplementary material for: Systematic analysis of lysine malonylation in Streptococcus mutans
Source: Front Cell Infect Microbiol. 2022 Nov 28;12:1078572. doi: 10.3389/fcimb.2022.1078572 (PMC9742479; doi:10.3389/fcimb.2022.1078572)
Supplement: Supplementary file 1 [file DataSheet_1.docx]

Supplementary Material

# Supplementary Tables

**Supplementary Table 1**. Complete list of Kmal sites identified in PG and PG groups.

**Supplementary Table 2**. Highly interconnected PPI networks.

**Supplementary Table 3**. Differentially modified sites.

**Supplementary Table 4**. List of the Kac-only, Kmal-only and overlapping sites.

# Supplementary Figures

**Supplementary Figure 1**. Bar graph for the information of LC–MS/MS spectrum database search analysis.

**Supplementary Figure 2**. Functional enrichment-based cluster analysis of differentially modified proteins. (**A**) Number of sites those are divided into to four comparable groups based on modification fold change (FC). (**B**) GO and KEGG enrichment-based cluster analysis of differentially modified proteins.

**Data Availability Statement**

The MS proteomics data presented in the study are deposited in the PRIDE repository (https://www.ebi.ac.uk/pride), accession number PXD038045.
